# 1-(4-Nitro­phen­yl)-2-(3-phenyl­allyl­idene)hydrazine

**DOI:** 10.1107/S1600536808021429

**Published:** 2008-07-19

**Authors:** Feng-Yu Bao

**Affiliations:** a Department of Applied Chemistry, College of Sciences, Henan Agricultural University, Zhengzhou 450002, People’s Republic of China

## Abstract

In the title compound, C_15_H_13_N_3_O_2_, the nitro­benzene and benzene rings make a dihedral angle of 9.1 (2)°. The crystal structure is consolidated by inter­molecular N—H⋯O hydrogen bonds.

## Related literature

For related literature, see: Okabe *et al.* (1993 ▶).
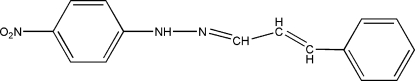


## Experimental

### 

#### Crystal data

C_15_H_13_N_3_O_2_
*M* *_r_* = 267.28
Monoclinic, 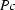

*a* = 6.1011 (3) Å
*b* = 12.4505 (6) Å
*c* = 9.0076 (4) Å
β = 93.273 (3)°
*V* = 683.12 (6) Å^3^
*Z* = 2
Mo *K*α radiation
μ = 0.09 mm^−1^
*T* = 296 (2) K
0.23 × 0.20 × 0.20 mm

#### Data collection

Bruker SMART APEX CCD area-detector diffractometer
Absorption correction: multi-scan (*SADABS*; Sheldrick, 1996 ▶) *T*_min_ = 0.980, *T*_max_ = 0.982
5992 measured reflections
1502 independent reflections
783 reflections with *I* > 2σ(*I*)
*R*_int_ = 0.045

#### Refinement

*R*[*F* ^2^ > 2σ(*F* ^2^)] = 0.039
*wR*(*F* ^2^) = 0.101
*S* = 0.94
1502 reflections
185 parameters
3 restraints
H atoms treated by a mixture of independent and constrained refinement
Δρ_max_ = 0.09 e Å^−3^
Δρ_min_ = −0.10 e Å^−3^

### 

Data collection: *SMART* (Bruker, 1998 ▶); cell refinement: *SAINT* (Bruker, 1998 ▶); data reduction: *SAINT*; program(s) used to solve structure: *SHELXS97* (Sheldrick, 2008 ▶); program(s) used to refine structure: *SHELXL97* (Sheldrick, 2008 ▶); molecular graphics: *SHELXTL* (Sheldrick, 2008 ▶); software used to prepare material for publication: *SHELXTL*.

## Supplementary Material

Crystal structure: contains datablocks global, I. DOI: 10.1107/S1600536808021429/hg2421sup1.cif

Structure factors: contains datablocks I. DOI: 10.1107/S1600536808021429/hg2421Isup2.hkl

Additional supplementary materials:  crystallographic information; 3D view; checkCIF report

## Figures and Tables

**Table 1:** Hydrogen-bond geometry (Å, °)

*D*—H⋯*A*	*D*—H	H⋯*A*	*D*⋯*A*	*D*—H⋯*A*
N2—H2*A*⋯O2^i^	0.89 (3)	2.20 (3)	3.082 (4)	169 (3)

Symmetry code: (i) 
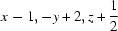
.

**Fig. 1.**
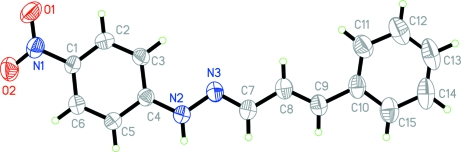
the ORTEP plot of (I). Displacement ellipsoids are drawn at the 30% probability level. H atoms are presented as small spheres of arbitrary radii.

**Fig. 2.**
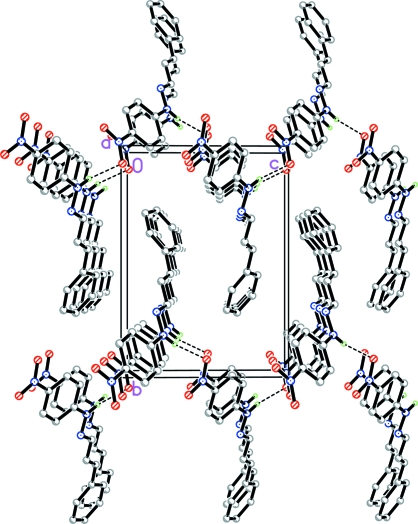
Packing of (I), showing the intermolecular hydrogen bonds as dashed lines.

## supplementary crystallographic information

### Comment

4-Nitrophenylhydrazine has applications in organic synthesis and some of its derivatives have been shown to be potentially DNA-damaging and mutagenic agents (Okabe *et al.*, 1993). As part of our interest in the study of the coordination chemistry, we report the synthesis and crystal structure of the title compound (I).

The 4-nitrophenyl group and the benzene ring are slightly twisted, making a dihedral angle of 9.1 (2)°. The dihedral angle between the N1/O1/O2 and its attached benzene ring is 7.4 (2)° (Fig. 1).

The molecules are linked by N—H···O hydrogen bonds to form a zigzag like chain (Fig. 2).

### Experimental

4-Nitrophenylhydrazine (1 mmol, 0.153 g) was dissolved in anhydrous methanol and H_2_SO_4_ (98% 0.5 ml) was added.  The resultant solution was stirred for several minutes at 351 K. Cinnamaldehyde (1 mmol 0.132 g) in methanol (8 ml) was added dropwise and the mixture was stirred at refluxing temperature for 2 h. The product was isolated and recrystallized in dichloromethane. Brown single crystals of (I) were obtained after 6 d.

### Refinement

All H atoms were placed in calculated positions, with C—H = 0.93Å (aromatic) with *U*_iso_(H) = 1.2*U*_eq_(C). H2A was located from a difference Fourier map and refined isotropically, with N–H distance restrained to 0.89 (3)Å.

### Crystal data

**Table:**

C_15_H_13_N_3_O_2_	*F*_000_ = 280
*M**_r_* = 267.28	*D*_x_ = 1.299 Mg m^−^^3^
Monoclinic, *P**c*	Mo *K*α radiation λ = 0.71073 Å
Hall symbol: P -2yc	Cell parameters from 1143 reflections
*a* = 6.1011 (3) Å	θ = 2.1–25.6º
*b* = 12.4505 (6) Å	µ = 0.09 mm^−^^1^
*c* = 9.0076 (4) Å	*T* = 296 (2) K
β = 93.273 (3)º	Block, brown
*V* = 683.12 (6) Å^3^	0.23 × 0.20 × 0.20 mm
*Z* = 2	

### Data collection

**Table:**

Bruker SMART APEX CCD area-detector diffractometer	1502 independent reflections
Radiation source: sealed tube	783 reflections with *I* > 2σ(*I*)
Monochromator: graphite	*R*_int_ = 0.045
*T* = 296(2) K	θ_max_ = 27.0º
φ and ω scans	θ_min_ = 1.6º
Absorption correction: multi-scan(SADABS; Sheldrick, 1996)	*h* = −7→7
*T*_min_ = 0.980, *T*_max_ = 0.982	*k* = −14→15
5992 measured reflections	*l* = −11→11

### Refinement

**Table:**

Refinement on *F*^2^	Secondary atom site location: difference Fourier map
Least-squares matrix: full	Hydrogen site location: inferred from neighbouring sites
*R*[*F*^2^ > 2σ(*F*^2^)] = 0.039	H atoms treated by a mixture of independent and constrained refinement
*wR*(*F*^2^) = 0.101	*w* = 1/[σ^2^(*F*_o_^2^) + (0.0488*P*)^2^] where *P* = (*F*_o_^2^ + 2*F*_c_^2^)/3
*S* = 0.95	(Δ/σ)_max_ < 0.001
1502 reflections	Δρ_max_ = 0.09 e Å^−^^3^
185 parameters	Δρ_min_ = −0.10 e Å^−^^3^
3 restraints	Extinction correction: SHELXL97 (Sheldrick, 2008), Fc^*^=kFc[1+0.001xFc^2^λ^3^/sin(2θ)]^-1/4^
Primary atom site location: structure-invariant direct methods	Extinction coefficient: 0.027 (6)

### Special details

**Table:**

Geometry. All e.s.d.'s (except the e.s.d. in the dihedral angle between two l.s. planes) are estimated using the full covariance matrix. The cell e.s.d.'s are taken into account individually in the estimation of e.s.d.'s in distances, angles and torsion angles; correlations between e.s.d.'s in cell parameters are only used when they are defined by crystal symmetry. An approximate (isotropic) treatment of cell e.s.d.'s is used for estimating e.s.d.'s involving l.s. planes.
Refinement. Refinement of *F*^2^ against ALL reflections. The weighted *R*-factor *wR* and goodness of fit *S* are based on *F*^2^, conventional *R*-factors *R* are based on *F*, with *F* set to zero for negative *F*^2^. The threshold expression of *F*^2^ > σ(*F*^2^) is used only for calculating *R*-factors(gt) *etc*. and is not relevant to the choice of reflections for refinement. *R*-factors based on *F*^2^ are statistically about twice as large as those based on *F*, and *R*- factors based on ALL data will be even larger.

### Fractional atomic coordinates and isotropic or equivalent isotropic displacement parameters (Å^2^)

**Table:**

	*x*	*y*	*z*	*U*_iso_*/*U*_eq_	
O1	1.0954 (5)	0.9273 (2)	−0.0961 (4)	0.1019 (9)	
O2	1.0374 (5)	1.0780 (3)	0.0100 (3)	0.1053 (11)	
N1	0.9922 (5)	0.9832 (3)	−0.0132 (3)	0.0768 (9)	
N2	0.2734 (4)	0.8124 (2)	0.2572 (3)	0.0689 (8)	
N3	0.1907 (4)	0.7139 (2)	0.2171 (3)	0.0718 (8)	
C1	0.8094 (5)	0.9377 (3)	0.0590 (4)	0.0595 (8)	
C2	0.7374 (5)	0.8364 (3)	0.0209 (4)	0.0663 (10)	
H2	0.8096	0.7967	−0.0488	0.080*	
C3	0.5603 (5)	0.7940 (3)	0.0852 (4)	0.0644 (9)	
H3	0.5116	0.7255	0.0585	0.077*	
C4	0.4516 (5)	0.8519 (3)	0.1903 (4)	0.0593 (8)	
C5	0.5270 (6)	0.9545 (3)	0.2290 (4)	0.0689 (10)	
H5	0.4567	0.9941	0.2998	0.083*	
C6	0.7040 (5)	0.9972 (3)	0.1631 (4)	0.0695 (9)	
H6	0.7532	1.0659	0.1882	0.083*	
C7	0.0126 (6)	0.6853 (3)	0.2753 (4)	0.0717 (9)	
H7	−0.0511	0.7305	0.3429	0.086*	
C8	−0.0884 (6)	0.5852 (3)	0.2375 (4)	0.0742 (10)	
H8	−0.0170	0.5393	0.1747	0.089*	
C9	−0.2781 (6)	0.5539 (3)	0.2868 (4)	0.0757 (10)	
H9	−0.3443	0.6017	0.3497	0.091*	
C10	−0.3973 (6)	0.4540 (3)	0.2553 (4)	0.0781 (11)	
C11	−0.3260 (8)	0.3749 (4)	0.1614 (5)	0.0973 (13)	
H11	−0.1919	0.3827	0.1184	0.117*	
C12	−0.4506 (11)	0.2854 (4)	0.1313 (6)	0.1188 (19)	
H12	−0.4012	0.2338	0.0664	0.143*	
C13	−0.6461 (12)	0.2703 (5)	0.1945 (7)	0.128 (2)	
H13	−0.7300	0.2094	0.1724	0.153*	
C14	−0.7170 (8)	0.3456 (5)	0.2905 (7)	0.1205 (19)	
H14	−0.8479	0.3346	0.3363	0.145*	
C15	−0.5968 (7)	0.4385 (4)	0.3210 (5)	0.0968 (13)	
H15	−0.6488	0.4901	0.3849	0.116*	
H2A	0.205 (5)	0.852 (2)	0.323 (3)	0.080*	

### Atomic displacement parameters (Å^2^)

**Table:**

	*U*^11^	*U*^22^	*U*^33^	*U*^12^	*U*^13^	*U*^23^
O1	0.0786 (17)	0.127 (2)	0.1032 (19)	−0.0004 (17)	0.0351 (16)	−0.0009 (18)
O2	0.104 (2)	0.104 (2)	0.109 (2)	−0.040 (2)	0.0197 (18)	−0.0008 (18)
N1	0.068 (2)	0.094 (3)	0.0691 (19)	−0.012 (2)	0.0059 (17)	0.008 (2)
N2	0.0610 (19)	0.070 (2)	0.077 (2)	−0.0064 (16)	0.0103 (16)	−0.0075 (15)
N3	0.0674 (19)	0.0658 (19)	0.082 (2)	−0.0096 (16)	0.0027 (17)	0.0023 (16)
C1	0.0512 (18)	0.070 (2)	0.0578 (19)	−0.0043 (18)	0.0033 (16)	0.0044 (17)
C2	0.069 (2)	0.069 (2)	0.062 (2)	0.0083 (19)	0.0106 (19)	−0.0015 (18)
C3	0.062 (2)	0.055 (2)	0.076 (2)	0.0008 (18)	0.0037 (19)	−0.0048 (19)
C4	0.0539 (19)	0.064 (2)	0.0598 (19)	−0.0012 (17)	0.0007 (17)	0.0004 (17)
C5	0.061 (2)	0.072 (2)	0.074 (2)	−0.0022 (18)	0.0110 (19)	−0.0102 (19)
C6	0.067 (2)	0.065 (2)	0.076 (2)	−0.011 (2)	0.0021 (19)	−0.0015 (19)
C7	0.062 (2)	0.075 (2)	0.077 (2)	−0.007 (2)	0.0044 (19)	0.0044 (19)
C8	0.074 (2)	0.068 (2)	0.080 (3)	−0.008 (2)	0.000 (2)	0.0086 (19)
C9	0.077 (3)	0.074 (2)	0.076 (2)	−0.016 (2)	−0.003 (2)	0.0047 (19)
C10	0.073 (3)	0.080 (3)	0.079 (3)	−0.018 (2)	−0.014 (2)	0.018 (2)
C11	0.123 (3)	0.086 (3)	0.082 (3)	−0.024 (3)	−0.002 (3)	−0.001 (2)
C12	0.169 (6)	0.082 (3)	0.102 (4)	−0.034 (4)	−0.022 (4)	0.011 (3)
C13	0.157 (6)	0.098 (4)	0.122 (4)	−0.061 (4)	−0.042 (4)	0.025 (4)
C14	0.096 (3)	0.119 (4)	0.144 (5)	−0.045 (4)	−0.022 (3)	0.045 (4)
C15	0.077 (3)	0.093 (3)	0.119 (3)	−0.014 (3)	−0.009 (3)	0.019 (2)

### Geometric parameters (Å, °)

**Table:**

O1—N1	1.221 (4)	C7—C8	1.423 (5)
O2—N1	1.228 (4)	C7—H7	0.9300
N1—C1	1.438 (4)	C8—C9	1.322 (5)
N2—C4	1.365 (4)	C8—H8	0.9300
N2—N3	1.366 (4)	C9—C10	1.461 (5)
N2—H2A	0.89 (3)	C9—H9	0.9300
N3—C7	1.284 (4)	C10—C11	1.384 (6)
C1—C2	1.373 (4)	C10—C15	1.397 (5)
C1—C6	1.382 (4)	C11—C12	1.368 (6)
C2—C3	1.361 (4)	C11—H11	0.9300
C2—H2	0.9300	C12—C13	1.364 (8)
C3—C4	1.388 (4)	C12—H12	0.9300
C3—H3	0.9300	C13—C14	1.362 (8)
C4—C5	1.396 (4)	C13—H13	0.9300
C5—C6	1.369 (4)	C14—C15	1.389 (7)
C5—H5	0.9300	C14—H14	0.9300
C6—H6	0.9300	C15—H15	0.9300
			
O1—N1—O2	122.2 (3)	N3—C7—H7	119.7
O1—N1—C1	119.5 (4)	C8—C7—H7	119.7
O2—N1—C1	118.3 (3)	C9—C8—C7	123.6 (4)
C4—N2—N3	119.9 (3)	C9—C8—H8	118.2
C4—N2—H2A	120 (2)	C7—C8—H8	118.2
N3—N2—H2A	120 (2)	C8—C9—C10	128.4 (4)
C7—N3—N2	116.7 (3)	C8—C9—H9	115.8
C2—C1—C6	120.5 (3)	C10—C9—H9	115.8
C2—C1—N1	119.6 (3)	C11—C10—C15	118.2 (4)
C6—C1—N1	119.9 (3)	C11—C10—C9	123.7 (4)
C3—C2—C1	120.1 (3)	C15—C10—C9	118.1 (4)
C3—C2—H2	120.0	C12—C11—C10	120.7 (5)
C1—C2—H2	120.0	C12—C11—H11	119.6
C2—C3—C4	120.7 (3)	C10—C11—H11	119.6
C2—C3—H3	119.7	C13—C12—C11	121.2 (5)
C4—C3—H3	119.7	C13—C12—H12	119.4
N2—C4—C3	122.5 (3)	C11—C12—H12	119.4
N2—C4—C5	118.6 (3)	C14—C13—C12	119.2 (5)
C3—C4—C5	118.8 (3)	C14—C13—H13	120.4
C6—C5—C4	120.3 (3)	C12—C13—H13	120.4
C6—C5—H5	119.9	C13—C14—C15	121.0 (6)
C4—C5—H5	119.9	C13—C14—H14	119.5
C5—C6—C1	119.7 (3)	C15—C14—H14	119.5
C5—C6—H6	120.2	C14—C15—C10	119.6 (5)
C1—C6—H6	120.2	C14—C15—H15	120.2
N3—C7—C8	120.7 (4)	C10—C15—H15	120.2

### Hydrogen-bond geometry (Å, °)

**Table:**

*D*—H···*A*	*D*—H	H···*A*	*D*···*A*	*D*—H···*A*
N2—H2A···O2^i^	0.89 (3)	2.20 (3)	3.082 (4)	169 (3)

Symmetry codes: (i) *x*−1, −*y*+2, *z*+1/2.
